# Diversity and Species Composition of Midgut Symbiotic Bacteria in *Culex quinquefasciatus* Mosquitoes in Gampaha District, Sri Lanka

**DOI:** 10.1155/2024/1832200

**Published:** 2024-07-25

**Authors:** Randi Hathnagoda, Pinidi Gunathilake, Thilini Buddhinee, Pabasara Welgama, Hasini Gunarathna, Harshani Perera, Koshila Ranasinghe

**Affiliations:** ^1^Department of Biomedical Sciences, Faculty of Health Sciences, CINEC Campus, Malabe, Sri Lanka; ^2^Department of Zoology and Environmental Management, Faculty of Science, University of Kelaniya, Dalugama, Kelaniya GQ 11600, Sri Lanka

## Abstract

Mosquitoes, notorious for their deadly impact as disease vectors, also hold economic value owing to their roles in disease transmission. The present study focuses on the importance of understanding mosquito gut microbiota for implementing innovative vector control strategies, thereby mitigating disease transmission. The study was conducted in the Gampaha Medical Office of Health (MOH) area of Sri Lanka with the focus of elucidating the microbial diversity within the midgut of *Culex quinquefasciatus*, a crucial step to support ongoing paratransgenesis efforts. Sampling was performed by utilizing standard mosquito sampling techniques and their midgut homogenates were plated on Plate Count Agar to isolate bacteria, which were then identified through biochemical tests. Subsequently, the most abundant bacterial families were subjected to DNA extraction, PCR amplification, and gene sequencing for species identification. The study revealed the presence of four bacterial families (Staphylococcaceae, Streptococcaceae, Neisseriaceae, and Moraxellaceae) in adult mosquitoes, while larvae harbored an additional family, Micrococcaceae. Interestingly, the relative distribution of midgut bacteria varied significantly among field-caught larval and adult strains from different study areas (chi-square = 1.673; *P* < 0.05), indicating similar bacterial flora across mosquito life stages and geographical locations. Of particular interest is the identification of *Lysinibacillus sphaericus*, a bacterium with potential for paratransgenesis applications. Given the high mosquito density in the study area, leveraging paratransgenesis for *Cx. quinquefasciatus* control is recommended. Furthermore, insights into gut microbes could inform the integration of gut microflora from modified strains into existing Sterile Insect Technique (SIT) and Incompatible Insect Technique (IIT) approaches in Sri Lanka.

## 1. Introduction

Mosquitoes are considered the world's deadliest arthropod animals as they are in charge of the transmission of many medically important pathogens [1, 2]. They transmit pathogenic bacteria, viruses, protozoans, and nematodes which cause serious human diseases such as malaria, dengue, chikungunya, Zika, West Nile, yellow fever, encephalitis, or filariasis [3–6]. Maintenance of the ecosystem functions of mosquitoes while reducing disease burden is a must. Targeting specific species or making the mosquitoes themselves immune to pathogens and thus the inability of them to spread would protect humans while keeping the ecosystem function of mosquitoes intact. Though there are different ways to take mosquito control measures, outwitting mosquitoes has become a challenging task due to their complex life cycle [7].

The variability within urban environments creates a complex landscape where mosquito populations and the spread of diseases they carry, such as West Nile, dengue, and Zika viruses, are influenced by a combination of social and ecological factors [8]. These dynamics have significant consequences for public health, as highlighted by recent research [9]. Each year, nations experience billions of dollars in losses due to investments in treating mosquito-borne diseases. For instance, households bear a substantial burden, with an estimated 25% of their income being spent on out-of-pocket expenses (OOPE) for malaria treatment, travel associated with treatment, and other diagnosis-related costs [10]. In many low- and middle-income countries (LMICs), urban areas with dense populations often suffer from poor hygiene and water quality, creating ideal conditions for increased mosquito breeding and the rapid spread of mosquito-borne diseases. Additionally, due to limited public health investments, most LMICs lack universal health coverage (UHC), leaving a large proportion of the population vulnerable to out-of-pocket expenses (OOPE) for healthcare. This situation can lead to catastrophic health expenditures for some individuals, pushing them into poverty [11].

Mosquito-borne diseases pose a significant threat to livestock health. In Florida, where mosquitoes are vectors for diseases like Eastern Equine Encephalitis (EEE) and West Nile Virus, livestock health faces substantial challenges. Livestock, including cattle, horses, and sheep, are vulnerable to these diseases leading to illness, reduced productivity, and in severe cases finally death. The economic losses incurred due to veterinary care, loss of livestock, and decreased productivity can be staggering for farmers and the industry as a whole. Additionally, the stress and discomfort caused by mosquito bites can further exacerbate the situation, leading to reduced feed intake, weight loss, and lower milk production in dairy cattle. These challenges highlight the critical importance of effective mosquito control strategies in safeguarding livestock health [12].

Filariasis is a significant social and health concern across Asian countries, often accompanied by stigma and widespread morbidity. Mosquitoes not only act as carriers of diseases but also bring about considerable nuisance. Tropical regions, in particular, bear the brunt of both disease prevalence and mosquito annoyance. Among the mosquito genus *Culex*, *Culex quinquefasciatus* stands out as a highly anthropophilic species, playing a major role in transmitting filariasis [13].

Surveillance activities carried out to evaluate the Program for Elimination of Lymphatic Filariasis (PELF) in Sri Lanka revealed the sporadic occurrence of brugian filariasis [14, 15] of probable zoonotic origin [16] in endemic areas. In 2016, Sri Lanka was certified to have eliminated lymphatic filariasis as a public health problem by the World Health Organization [17]. However, the occurrence of zoonotic brugian filariasis raises an issue as mass treatment of the human population with anti-filarial drugs would not eliminate the reservoir of infection nor the vector population. Human dirofilariasis is another zoonotic filarial infection, commonly encountered in the country [18–20].

The abundance of disease-transmitting vectors due to limited vector control activities and higher receptivity in these areas have been identified as major challenges to maintaining the disease-free status in the country, for filariasis. Therefore, alternative vector control methods are needed as additional tools to be incorporated into integrated vector management [19].

In recent years, scientists have investigated the potential use of symbiotic bacteria in mosquitoes for vector control interventions [21]. In blood-feeding insect vectors, microbiota plays another vital function by affecting the vectorial competence ability to transmit pathogens to their target species. Monitoring the gut bacteria in a genetically altered insect strain offers a fresh perspective on vector competence and fitness. This is because the types of bacteria present in the gut can impact the insect's immunity. By keeping an eye on these bacteria, we can better understand how they influence the insect's ability to transmit diseases and their overall health and vigor [21]. Hence, evaluation of the gut microbial community in the wild population where the novel vector control approaches have been targeted is of paramount importance to get an idea of the vectorial potential and fitness. Furthermore, screening gut microbes would be beneficial in identifying the potential microbial candidates and exploring the feasibility of using some of these bacteria for novel vector control strategies such as paratransgenesis.

Besides, the diversity of the gut flora of filaria-transmitting vector mosquitoes, *Cx. quinquefasciatus*, in Sri Lanka has rarely been studied although some detailed studies have been made only to screen *Wolbachia* strain in mosquitoes and studies have been conducted to identify gut flora of *Culex tritaeniorhynchus* and *Culex gelidus* mosquitoes only in Gampaha District, Sri Lanka [22]. Hence, the present study analyzed and compared the occurrence of gut microbial bacteria among larval and adult stages of the filariasis vector, *Culex quinquefasciatus*, as a fundamental requirement to support the SIT and IIT approaches which are in progress in Sri Lanka.

## 2. Methodology

### 2.1. Selection of Study Area

Mosquito surveys were conducted in three areas: Kelaniya (6.9465° N, 79.9024° E), Gampaha (6.5924° N, 79.5247° E), and Meerigama (7.0905° N, 80.0556°), in the Gampaha Medical Office of Health (MOH) area, Gampaha District, Western Province of Sri Lanka. This district has been identified and surveyed for vector mosquitoes systematically targeting to implement the SIT-based control approaches using irradiated males and locally developed strains of *Wolbachia* triple-infected mosquitoes. Therefore, surveillance areas for mosquito collections were purposely selected from the Gampaha District, to provide fundamental evidence required for some novel vector control interventions.

## 3. Field Collection of Mosquitoes

**Figure 1:**
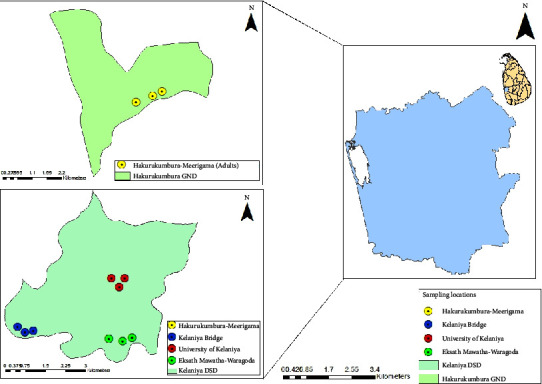
Sampling locations in the study area.

Sampling was performed bimonthly from September 2022 to December 2022. Three mosquito breeding sites were selected within the Gampaha District, and each sampling site was geo-referenced (GARMIN-etrex SUMMIT) (Figure 1).

Adult mosquitoes were collected using the Prokopak apparatus (John W. Hock Company, Gainesville, FL, USA) at outdoor resting sites. The larvae were collected by the siphoning/pipetting method from container breeding habitats following the guidelines described by the World Health Organization [23].

Collected adult mosquitoes were transferred safely to the presterilized adult rearing cages while the collected larvae were transferred safely into larval rearing containers. All collected larval and adult mosquito samples were transported to the laboratory facility at the Department of Biomedical Sciences, Faculty of Health Sciences, Colombo International Nautical and Engineering College (CINEC), Sri Lanka, and kept alive until use for the experiment.

*Cx. quinquefasciatus* labeled for mosquito species identification and larvae in each sample were identified into species level using standard identification keys [24–27] and observed under the light microscope (Euromex, China)

### 3.1. Processing of Mosquito Samples

Adult mosquitoes (adult and larvae) were killed using a cold shock followed by separation based on key morphological characteristics. Larvae of stages III and IV were taken for the experiment. Specifically, stages III and IV of the larvae were selected because their morphological characteristics are well developed and distinct making them more suitable for the studying aspect. The last instar stages have a well-established midgut symbiotic bacterial diversity since their feeding period in water is longer compared to 1^st^ instar or 2^nd^ instar stages. Furthermore, from the collected adults, only adult females were used for the experiment. The specimens were surface-sterilized individually for 30 seconds in a microcentrifuge tube containing 250 *μ*L of 70% ethanol followed by rinsing twice with 250 *μ*L of sterilized phosphate-buffer saline (PBS). The final discard was taken for bacteria screening to make sure that there was no contamination of transient flora.

The midgut of the adult female mosquitoes and larvae (stage III-IV) was dissected under sterile conditions. The sterile protocol was followed throughout. Before dissection, all instruments and reagents used are typically sterilized using methods such as autoclaving, dry heat sterilization, and soaking in alcohol to minimize sterilization. The dissection area was set up in a sterile clean bench. The workspace was cleaned thoroughly with 70% ethanol and sterile equipment and reagents were used throughout the procedure. Adult female mosquitoes and the selected larvae were immobilized with cold shock before dissection, and when using sterile instruments, the mosquitoes were immobilized and positioned under a dissecting microscope. The abdomen was carefully opened, and the midgut was located and dissected out from the surrounding tissues. Special care was taken to avoid contamination from other tissues or organs. Similarly, larvae at stage III-IV were typically dissected by immobilizing them and carefully removing the outer cuticle layers to expose the midgut. The dissection is performed under a microscope to ensure precision and minimize damage to the tissue. Throughout the procedure, strict sterile techniques were maintained to minimize contamination from external sources. This included using sterile tools, working in a sterile environment, and avoiding contact with nonsterile surfaces.

Individually dissected midgut of the adult/larvae of each mosquito was transferred to a 1.5 mL microcentrifuge tube containing 250 *μ*L of PBS and homogenized with a sterilized micropestle. The homogenized lysate was serially diluted in PBS (900 *μ*L) to prepare a dilution series ranging from 10^−1^ to 10^−7^. A minimum sample size of 300 adults and larvae was screened for lumen bacteria in each field collected from *Cx. quinquefasciatus* mosquitoes.

### 3.2. Culturing and Isolation of Bacteria from Microbiological Techniques

A volume of 100 *μ*L from each dilution was plated on sterile Plate Count Agar (PCA) media and incubated at 35°C–37°C for 24–48 hours. The microbial growth was assessed through the total number of colony forming units (CFUs). Bacterial colonies were distinguished morphologically (shape, size, color, margin, opacity, elevation, etc.). Morphologically distinct colonies were selected from primary plates for repeated subculture on nutrient agar (NA) plates using the streak plate technique until they obtained a pure colony. All bacterial isolates were identified up to the genus level by Gram staining and biochemical testing performed according to Cowan and Steel's manual [28, 29]. Stab cultures of each bacteria were prepared in a slant of NA medium in Eppendorf tubes (1 mL). Glycerol stocks of each isolated bacteria were made for long-term storage of the bacterial species.

### 3.3. Preparation of Glycerol Stocks for Long-Term Storage of Bacterial Cells at −80°C

Sterile (autoclaved) 50% glycerol solution in Aqua dest was used for this. As glycerol is rather viscous, stock glycerol was poured directly into a bottle, and the volume was estimated with the naked eye along the volume scale. Before aliquoting the 50% glycerol solution, a magnetic bar was added and the solution was heated on a magnetic stirrer. After heating, the solution was easily pipetted and aliquoted into cryo tubes with screw caps as follows. Solution was autoclaved. Freshly grown bacteria on nutrient agar plates were taken and the biomass was scraped with a sterile inoculation loop. They were separately dissolved in a sterile liquid medium of nutrient agar broth to be grown. Samples were labeled properly to avoid the risk of getting thrown away when freezing. Then test tubes were vortexed and simply aliquot aseptically into sterile cryo vials (2.5 mL) with 50% glycerol. For cryotubes with 300 *μ*l 50% glycerol solution, a 700 *μ*l liquid broth sample was added and then carefully vortexed. Vortexed cryo vials were placed at −80°C. Triplicates or more were prepared for each bacterial isolate and they were stored in separate boxes.

### 3.4. Biochemical Tests for Identification of Bacteria up to Genus Level

Several biochemical tests were performed for the identification of bacterial isolates as per identification keys [28]. Oxidase Test (OXI), Catalase Test (CAT), Gram's Staining, Citrate Utilization Test (CIT), Kligler's Iron Agar Test (KIA), Sulfide Indole Motility Test (SIM), Bile Esculin Test, Methyl Red Test (MR), and Voges–Proskauer Test (VP) were performed.

### 3.5. Confirmation of Bacterial Isolates from Genomic Sequencing

#### 3.5.1. Bacterial DNA Extraction

Bacterial DNA was extracted from pure cultures using the CEYGEN Geno Spin D™ Genomic DNA extraction kit (CEYGEN Biotech (Pvt) Ltd., Sri Lanka) according to the manufacturer's instructions. In brief, selected stab cultures were grown on culture plates to obtain pure colonies. Bacterial samples were prepared by dissolving 2-3 loopfuls of each colony in 300 *μ*l of PBS in microcentrifuge tubes (1.5 mL). DNA extraction was performed by adding binding buffer, Proteinase K, and the prepared microbial sample to microcentrifuge tubes, followed by incubation at 56°C for 10 minutes. After centrifugation, isopropanol was added, and the mixture was transferred to columns for further processing. Wash buffers were added sequentially, and DNA was eluted with elution buffer.

#### 3.5.2. Agarose Gel Electrophoresis

A gel electrophoresis was performed to check whether the DNA of each bacterial colony was extracted efficiently. For that, the gel was prepared, and it was placed in the chamber covered with a TBA buffer. Loading dye (5 *μ*L) and the extracted products (5 *μ*L) were added to each well. The DNA ladder (0.5 *μ*L) was added as well. 60 V of voltage was supplied for 20–30 minutes and the products were visualized under the UV light using the UV transilluminator.

#### 3.5.3. The 16S rRNA PCR Amplifications

The 16S rRNA PCR amplifications were performed using universal primers 27F (5′ AGAGTTTGATCCTGGCTCAG 3) and 1492R (5′ TACGGCTACCTTGTTACGACTT 3′) [30]. Polymerase Chain Reaction (PCR) was performed with a reaction mixture containing 1x PCR buffer (Invitrogen), 0.5 *μ*M of each primer, 2.5 mM MgCl2, 200 ng of purified DNA, 0.2 mM dNTPs, and 0.3 units of Taq polymerase (Invitrogen). The total volume was adjusted to 25 *μ*L. The PCA media and doubled distilled H2O were used as negative controls.

The PCR was programmed for initial denaturation at 94°C for 10 min (2.5), followed by 35 cycles of denaturation at 94°C for 30 s, annealing at 55°C for 30 s, and extension at 72°C for 1 min. The final extension was at 72°C for 8 min. The amplified product was again visualized on a 1% agarose gel containing ethidium bromide using a UV transilluminator. The PCR amplicons were then purified using the QIAquick PCR Purification Kit (Qiagen). The purified products were sent to Macrogen, South Korea (Macrogen Inc., 1001, 254 Beotkkot-ro, Geumcheon-gu, Seoul, Republic of Korea), for 16S ribosomal RNA partial gene sequencing by the Sanger method.

### 3.6. Data Analysis

Homologous sequences were searched in the GenBank database using BLAST (http://www.ncbi.nlm.nih.gov/BLAST). The isolates were identified when their 16S rRNA sequences shared 97% homology with completed 16S rRNA sequences found in the GenBank database. Data were inserted into an MS Excel sheet for the preliminary statistical analysis for calculating the percentages and generation of graphs and tables.

### 3.7. Diversity of the Bacterial Community

The colony forming units (CFUs) for each identified bacteria were calculated using the following equation.

(1) $$\begin{array}{c} \frac{\text{CFU}}{\text{ml}}=\frac{\left(\text{No of colonies}\times \text{Total dilution factor}\right)}{\text{ The volume of culture plated in ml}}. \end{array}$$

Excel spreadsheet tool [31] was used to calculate confidence intervals for comparison of the presence of each bacterial species in mosquito species. The significance of the distribution of bacteria species in larval and adult *Cx. quinquefasciatus* was evaluated using the chi-square test. All the interpretations were done considering 95% significant intervals.

To highlight the differences in the midgut microbiota in larval and adult *Cx. quinquefasciatus* mosquitoes, the Distance-Based Redundancy Analysis (dbRDA) by Bray–Curtis (BC) dissimilarity [32] was used. To quantify the bacterial richness in each mosquito species obtained from the field, diversity indices such as Shannon and Weaver [33] and evenness [34] were used.

#### 3.7.1. Shannon Index

(2) $$\begin{array}{c} \text{Shannon Index }\left(H\right)=-\overset{x}{\underset{i=1}{\sum }}p_{i}\;\ln\;p_{i}. \end{array}$$

In the Shannon index, *p* is the proportion (*n*/*N*) of individuals of one particular species found (*n*) divided by the total number of individuals found (*N*), ln is the natural log, Σ is the sum of the calculations.

(3) $$\begin{array}{c} J=\frac{H^{\prime }}{H_{\max}}. \end{array}$$

#### 3.7.2. Pielou's Evenness (J)

Pielou's evenness compares the Shannon–Wiener diversity value (*H*′) to the maximum possible diversity value (*H*_max_).

## 4. Results

### 4.1. Diversity of Bacterial Isolates from Adult and Larval *Cx. quinquefasciatus*

**Figure 2:**
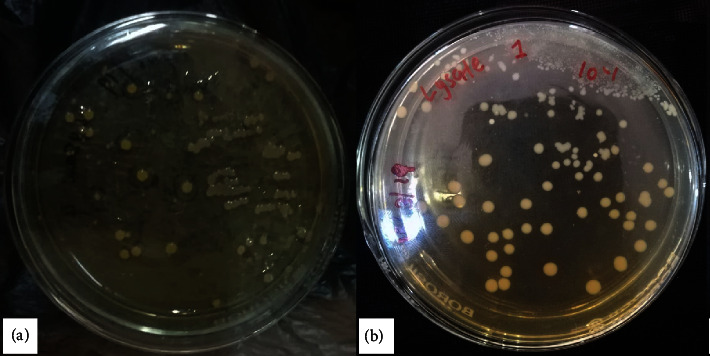
Primary culture plates of microbial colonies grown in nutrient agar medium at different dilutions: (a) 10^−2^ and (b) 10^−1^ of field-caught larval *Cx. quinquefasciatus*.

A total number of 12 isolates from mosquito adults and larvae were selected, and their colony morphology was examined (Table 1). There were 8 and 4 bacterial isolates from larvae and adults, respectively. Characteristics including elevation, size, form, surface appearance, color, margin, and opacity were used to identify the different bacterial colonies (Figure 2) (Table 1) and further classify them to the genera level. In terms of the number of bacterial colonies growing in the nutrient agar, a decreasing trend with increasing dilution of the culture medium in both adults and larvae of *Cx. quinquefasciatus* was observed. From the dilution series that was prepared, the best countable colonies were isolated in the range of 10^−3^ to 10^−5^ lysate dilutions of both larvae and adults.

### 4.2. Biochemical Characterization of Bacterial Isolates from *Cx. quinquefasciatus*

**Figure 3:**
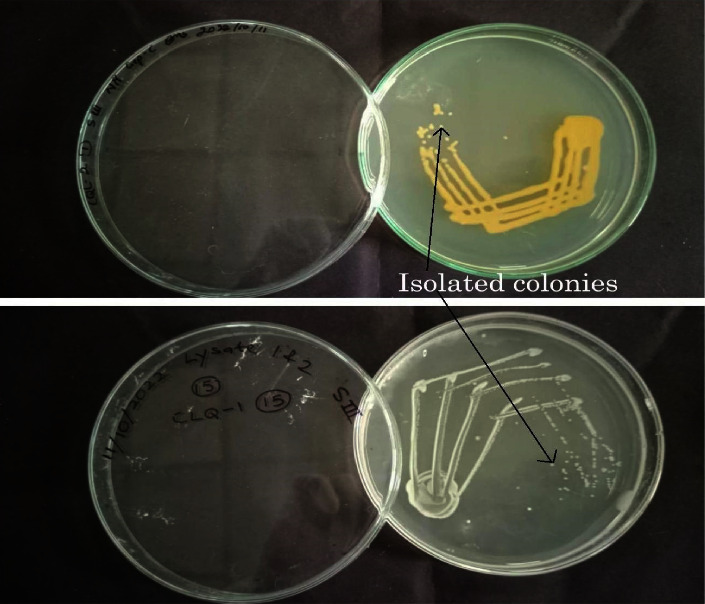
Single pure colonies isolated from the streak plate technique.

**Figure 4:**
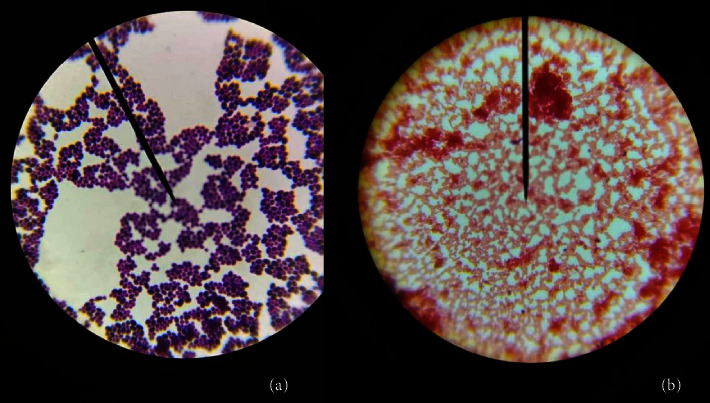
Microscopic view of (a) Gram-positive cocci and (b) Gram-negative cocci (X100).

**Figure 5:**
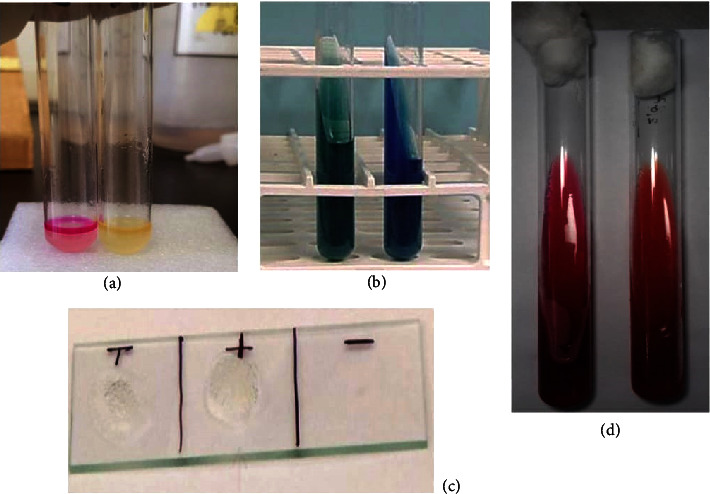
The positive and negative results of biochemical tests: (a) indole test; (b) citrate test; (c) catalase test; (d) TSI test.

The isolated pure colonies from the midgut of larval and adult *Cx. quinquefasciatus*, obtained using the streak plate technique (Figure 3), exhibited different results in the biochemical tests performed (Figures 4 and 5).

### 4.3. Bacterial Diversity in Field-Collected Adult and Larval Stages of *Cx. quinquefasciatus* Mosquitoes

Five bacterial strains *Staphylococcus* spp., *Streptococcus* spp., *Acinetobacter* spp., *Neisseria* spp., and *Micrococcus* spp. were identified from field-collected *Cx. quinquefasciatus* larvae and adults. *Micrococcus* spp. revealed the highest number of CFU per larva (Table 2). The bacterial community in the mosquito larvae stage midguts was dominated by 5 main genera: *Micrococcus, Streptococcus, Staphylococcus, Acinetobacter,* and *Neisseria*, while the midgut bacterial community in the adult stages was dominated by 4 major genera: *Staphylococcus, Streptococcus, Acinetobacter*, and *Neisseria*. In this diverse range of microbial communities, *Streptococcus* was the most abundant genera that existed.

**Figure 6:**
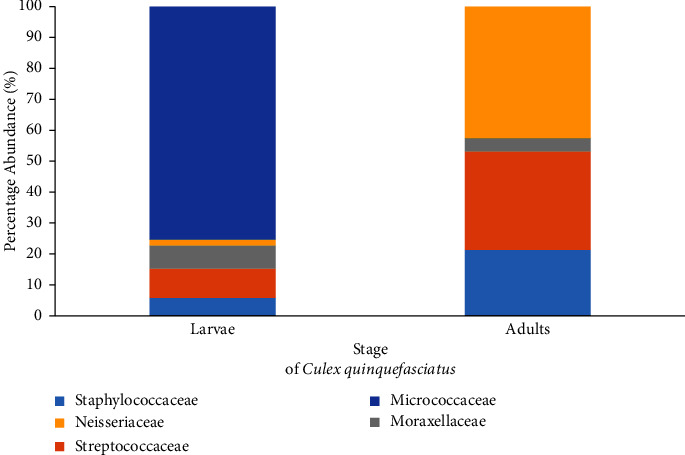
Relative abundance of bacterial families identified in the midgut of field-captured adult and larvae stages of *Cx. quinquefasciatus* mosquitoes.

The midgut bacteria identified in the field-collected larvae belonged to five families (Staphylococcaceae, Streptococcaceae, Neisseriaceae, Moraxellaceae, and Micrococcaceae). Study findings demonstrate that the aerobic microbial flora of the larvae mosquito midgut is complex compared to adults and is dominated by the Gram-positive Micrococcaceae (75%) family. Interestingly, Streptococcaceae (9%), Moraxellaceae (8%), and Staphylococcaceae (6%) families were also found but with fewer quantities (Figure 6). The least abundance of larval midgut bacterial composition was found in the family Neisseriaceae (2%). In contrast, the midgut bacterial composition of adult *Cx. quinquefasciatus* revealed that Neisseriaceae (43%) family was dominant. Moraxellaceae (4%) was found with the least abundance. Bacteria species belonging to Streptococcaceae (32%) and Staphylococcaceae (21%) families were found too (Figure 6).

Shannon diversity indices revealed that the gut microbiota of field-collected larval *Cx. quinquefasciatus* (1.48) was the most diverse. Evenness indices (0.92) also advocated the above observation.

### 4.4. Variation of Midgut Bacteria among Larval and Adult *Cx. quinquefasciatus*

Overall, the midgut bacteria of adult and larval *Cx. quinquefasciatus* stages indicated that there were no significant differences from each other (chi-square; *X*^2^ = 1.673; *P* < 0.05), revealing that both larvae and adults in two mosquito species harbor similar bacterial species as their gut microflora.

**Figure 7:**
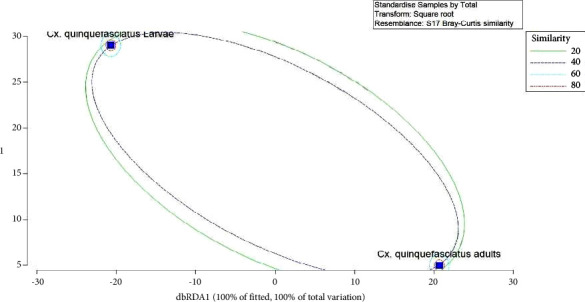
The distance-based redundancy analysis (dbRDA) plot for distribution of the midgut bacteria in the field-caught *Cx. quinquefasciatus* adults and larvae.

As indicated by the loadings of the dbRDA axes, the midgut bacteria in adult and larval *Cx. quinquefasciatus* indicated a similarity of 58.36%. According to the dbRDA, nearly 20.68% of variations in the midgut bacteria of the field caught *Cx. quinquefasciatus* (Figure 7) were explained. The dbRDA 1 axis was significantly influenced by the abundance of the *Streptococcus* genera.

### 4.5. Molecular Characterization of Midgut Bacteria of *Cx. quinquefasciatus*

**Figure 8:**
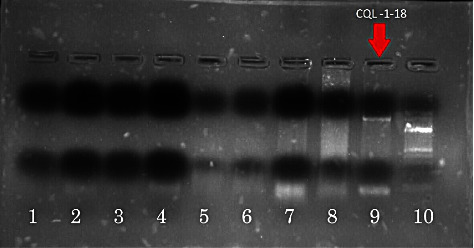
The gel picture shows PCR-amplified 16S rRNA gene products from midguts of field-collected *Cx. quinquefasciatus*. The gel image depicts the results of the PCR amplification of sample CQL-1-18 using universal primers 27F and 1492R (lane 10 : 50 bp DNA marker, lane 9: *Cx. quinquefasciatus* sample, lane 2: positive control, and lane 1: negative control).

**Figure 9:**
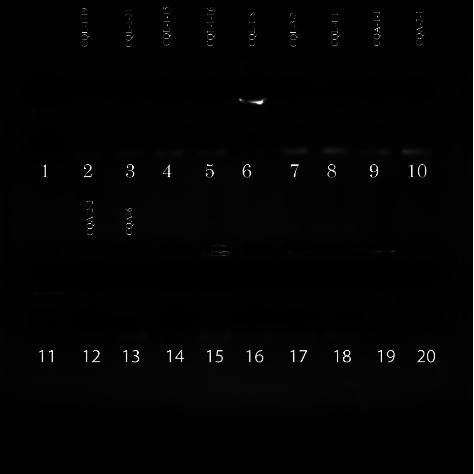
Gel picture showing PCR amplified 16S rRNA gene products from midguts of field-collected *Cx. quinquefasciatus*. The gel image depicts the results of the PCR amplification of samples 2 to 12 using universal primers 27F and 1492R (lanes 1 and 11 : 50 bp DNA marker, lane 2 to 10 and 12: *Cx. quinquefasciatus* samples, lane 13: positive control, and lane 15: negative control).

Amplification of 16S rRNA with bacterial universal primers 27F and 1492R yielded amplification products of approximately 1500 bp (Figures 8 and 9). Amplicons were then stained with ethidium bromide and visualized under UV on 1.5% agarose. The bands in the gel electrophoresis were of good quality and visible with better separation. Also, the separation of the PCR products and the DNA fragments was more than 1000 bp.

Gene sequencing revealed that the *Lysinibacillus sphaericus* was the most abundant microbial species isolated from the adults of midgut.

## 5. Discussion

The present study was carried out for the molecular characterization of culturable aerobic bacteria in the midgut of the filariasis vector, *Cx. quinquefasciatus*, in Gampaha District, Sri Lanka, to identify gut flora of larval and adult stages separately using entomological dissections, colony characteristics, biochemical testing, and DNA sequencing and to identify potential midgut bacteria in mosquitoes for the use of paratransgenesis control approaches. The bacteria inhabiting mosquito midgut have been targeted in the recent past due to their interactions with both mosquito hosts as well as disease-causing parasites. The present research generated descriptive information about aerobic bacterial flora in the midgut of the field-caught *Cx. quinquefasciatus* larvae and adult mosquitoes which was not studied in Sri Lanka so far. The richness and diversity of microbes associated with the field-collected larvae were found to be quite high in all the larval samples.

*Lysinibacillus sphaericus* belonging to the phylum Proteobacteria was also previously recorded as a potential candidate to control malaria transmission [35]. This species was recorded during the present study too, as the most dominant symbiotic bacteria from the gut of adult *Cx. quinquefasciatus*. Therefore, *Lysinibacillus sphaericus* emerges as a potential candidate to be used for a potential paratransgenesis approach in the future.

The mosquitoes' midgut bacteria play an important role in vector parasite interactions [36]. The present study area, Gampaha District, a highly urbanized region with the endemic transmission of filariasis, is dominated by this filarial vector mosquito *Cx. quinquefasciatus*. The prevalence of culturable bacteria in different populations of the same species and in different species of mosquitoes seems to be quite variable [37]. In the present study conducted, percent positivity for the presence of bacteria in any lysate ranged from 20% to 85% and maximum abundance and diversity of microbiota were recorded from *Cx. quinquefasciatus* population collected from the Kelaniya sampling area. Most of the species were unevenly distributed in the host gut.

Comparatively, the bacterial profile of larvae and adults of wild-captured *Cx. quinquefasciatus* was found quite dissimilar. However, *Staphylococcus, Streptococcus, Neisseria, and Acinetobacter* genera were found as common for both adults and larvae of wild-captured *Cx. quinquefasciatus*. As indicated by the loadings of dbRDA axes, the presence of *Streptococcus* sp. common to midgut microbiota in larval and adult *Cx. quinquefasciatus* has led to a share of similarity of 56.48% between stages. Therefore, it is evidenced that the midgut bacterial diversity is a reflection of bacteria acquired from various types of environments where the larvae or adults develop. Therefore, the midgut microbial diversity of mosquitoes is known to vary according to the life stage of mosquitoes [38].

The composition of bacterial communities varies according to different feeding regimes, and usually after a blood meal of females, the number of coexisting bacterial species becomes low due to interspecies competition between the bacterial isolates occurring in the midgut [39]. Therefore, when selecting female adults, unfed individuals were considered. Furthermore, the shifting of feeding habits in mosquitoes leads to a transformation of high carbohydrate levels to proteins, and that resulted in increased levels of enteric bacteria and a reduction of overall bacterial diversity [39, 40].

This study also proves that the microbial population can be inhibited in relation to different environmental conditions. Mosquitoes mainly acquire bacteria either from their larval habitat or from the environment during the process of nectar-feeding or blood-feeding [41]. Because of very low temperatures, environmental bacterial load becomes also very low which was also reflected in mosquito midgut bacterial diversity. Since many biotic and abiotic factors account for the distribution of microbes in the environment, other unidentified and ecologically important factors or interactions between these factors may account for the differences in richness observed across populations [42, 43]. Simultaneously, with the growth of the larvae, the prevailing microbial community can be minimized. As a result, the microbial population in the adults becomes less. Also, it indicates that many midgut microorganisms only grow in favour of water, and the aquatic environment consists of a variety of living organisms compared to the terrestrial environment [44]. That was further proven by the present study, in which the adults were found with less diversity of midgut bacteria.

Moreover, there were a large number of soil and environmental bacteria isolated in this study, such as *Acinetobacter* and *Micrococcus*. These isolates showed variations when compared with the stage of the life cycle of the mosquito *Cx. quinquefasciatus*. This suggests that the Sri Lankan soil and water environment plays an important role in the colonization of the mosquito midgut with regional bacteria encountered at breeding sites or during nectar or blood feeding.

When analyzing the genera according to the abundance of the prevailing microbes, there were common organisms for both larval and adult stages, such as *Streptococcus* and *Staphylococcus.* Some of the bacteria exhibited significant characteristics, such as the ability to inhibit physiological reactions, including DNA extraction, which was also observed during the present study.

Most of the genera recovered in this study have already been reported from the midgut of various mosquito vector species. All five bacterial genera have been previously reported to inhabit mosquito gut. However, it was the first time those genera were extracted in the larval stage of *Culex quinquefasciatus* mosquito. Members of genera *Acinetobacter, Aeromonas, Bacillus, Enterobacter, Enterococcus, Klebsiella, Pantoea, Pseudomonas, Serratia, Staphylococcus,* and *Stenotrophomonas* have been frequently reported from mosquito gut in previous studies and our results are consistent with those of the earlier reports [19, 22, 45–52].

This suggests that at least a part of the mosquito midgut microbial population could be common for different mosquito species inhabiting similar environments and may indicate evolutionary conservation of association between bacteria and larvae and adult mosquito gut. Bacterial isolates belonging to the genera *Streptococcus* and *Staphylococcus* were recovered from all the field locations and comprise a major part of the midgut microbiota of *Cx. quinquefasciatus* mosquitoes in the present study which is in accordance with earlier reports [37, 53]. It has been reported previously that species of *Enterobacter* are the most common bacteria isolated from insect gut [45, 54]. But interestingly during the present study, any *Enterobacter* was not isolated.

A previous study has indicated that *Pantoea agglomerans* could express and secrete anti-plasmodium effects or proteins (SM1, anti-Pbs21, and PLA2) [55]. Therefore, species in the genus *Pantoea* may be possible candidates for paratransgenesis [56]. Moreover, *Pantoea* exhibits transstadial and horizontal transmission properties [57]; thus, if used in the paratransgenesis approach, the modified bacteria could be introduced into the larval diet. This would allow the bacteria to be transmitted to the adult stages through both transstadial and horizontal transmission. A previous finding has confirmed that another species in the genus *Serratia*, namely, *S. marcescens* induces the mortality in *An. stephensi* when infected with *Plasmodium berghei* parasite [58]. However, during the present study, any species belonging to *Serratia* or *Pantoea* were not isolated from *Cx. quinquefasciatus*.

Many studies have summarized the positive and negative effects of these gut microbial communities on vector competency through interaction with hosts and parasites [36, 59]. Removal of the majority of midgut bacteria through antibiotic treatment results in greater susceptibility of *Ae. aegypti* to dengue virus (DENV) infection [36, 60]. Reintroduction of certain field-derived bacteria through a nectar meal into the mosquito gut increases the resistance to pathogen infection in *Ae. aegypti*, although not all bacteria tested influence the susceptibility to infection [61]. These studies highlight the importance of the interaction between the vector, microbiome, and the pathogen.

However, when using a culture-dependent approach, only 20% of environmental bacteria can be grown on a growth medium [62]. Thus, the composition of the microbiota is not a direct reflection of the richness and abundance of the whole gut bacterial community and rises as a limitation of the culture-dependent microflora analysis. The development of future perspectives for the paratransgenesis approach in Sri Lanka is interesting and necessary to examine the potential success of this method.

The present study arises as the first attempt at comparative analysis of midgut microbiota of larval and adult stages of *Culex quinquefasciatus*. Identification and characterization of mosquito midgut flora are likely to contribute towards a better understanding of mosquito biology including longevity, reproduction, and mosquito bacterial interaction and will be important to develop novel strategies for vector control. The use of gut microbacteria available in the insects for control approaches in the means of vector suppression, inhibition of parasites, pathogenic development in the invertebrate hosts, oviposition attractants in the development of lethal ovitraps, and transformation carriers in expressing molecules has been evaluated [63]. It is ideal to select nonpathogenic organisms for the proposed application of paratransgenesis.

## 6. Conclusions and Recommendations

Bacteria belonging to the genera *Streptococcus*, *Staphylococcus*, *Micrococcus*, *Neisseria,* and *Acinetobacter* were identified as midgut bacteria in larval *Cx. quinquefasciatus*. Similarly, bacteria from the genera *Streptococcus, Staphylococcus, Neisseria,* and *Acinetobacter* were identified as midgut bacteria of adult *Cx. quinquefasciatus.* The relative distribution of midgut microbes in different mosquito species was mostly similar among the larval and adult *Cx. quinquefasciatus* mosquitoes, revealing that both larvae and adults of different mosquito species harbor the same bacterial species as their midgut flora. *Lysinibacillus sphaericus* reported in the present study could be proposed as a potential candidate to be used in paratransgenesis. Since mosquito density is high in the present study area, the recommendation encourages control of *Cx. quinquefasciatus* through paratransgenesis. Furthermore, insights into gut microbes could be integrated with the gut microflora of modified strains produced through the current SIT and IIT approaches in Sri Lanka. This integration aims to create a modified organism that is compatible with the wild population, thereby enhancing the competitiveness, fitness, and survival of the modified population.

## Figures and Tables

**Table 1:** Colony morphology of selected isolates from the midgut of larvae and adult *Cx. quinquefasciatus*.

Strain	Form	Surface	Color	Margin	Elevation	Opacity	Identified bacteria
CQL 1	Circular	Smooth and glistening	Light orange (luminous)	Entire	Slightly raised	Opaque	*Staphylococcus* sp.
CQL 2	Circular	Rough and glistening	Pale yellow	Entire	Centrally raised	Transparent	*Streptococcus* sp.
CQL 3	Circular	Smooth and glistening	Bright yellow	Entire	Centrally raised	Opaque	*Staphylococcus* sp.
CQL 4	Circular	Smooth	Milky white	Entire	Centrally raised	Opaque	*Staphylococcus* sp.
CQL 5	Circular	Smooth and glistening	Light cream	Entire	Slightly flat	Translucent	*Neisseria* sp.
CQL 6	Circular	Smooth and glistening	Bright orange	Entire	Raised	Opaque	*Acinetobacter* sp.
CQL 7	Circular	Smooth, glistening, and mucoid	Light cream	Entire	Convex	Opaque	*Micrococcus* sp.
CQL 8	Circular	Smooth and glistening	Brick red	Entire	Centrally raised	Opaque	*Staphylococcus* spp.
CQA 1	Circular	Smooth and glistening	Milky white	Entire	Slightly flat	Translucent	*Streptococcus* sp.
CQA 2	Circular	Smooth	Cream	Entire	Slightly flat	Opaque	*Acinetobacter* sp.
CQA 3	Circular	Smooth	Cream	Entire	Slightly flat	Opaque	*Neisseria* sp.
CQA 4	Circular	Smooth	Cream	Entire	Centrally raised	Opaque	*Staphylococcus* sp.

CQL: *Culex quinquefasciatus* larvae; CQA: *Culex quinquefasciatus* adults.

**Table 2:** Colony forming unit (CFU) for identified bacteria in larval and adult stages of *Cx. quinquefasciatus*.

Colony number	Bacteria	Larvae		Adults	
		Counts per plate	CFU (ml)	Counts per plate	CFU (ml)
1	S*taphylococcus* spp.	40	400000	10	1000000
2	*Streptococcus* spp.	50	500000	150	1500000
3	*Acinetobacter s*pp.	40	400000	20	2000000
4	*Neisseria* spp.	10	100000	20	200000
5	*Micrococcus* spp.	70	7000000		

## Data Availability

The datasets supporting the conclusions of this article are included within the article. Data will not be shared in any of the sources.

## Additional Points

*Limitations*. During the present study, only selected bacteria were subjected to 16S rRNA gene sequencing due to financial limitations.
